# Identification and Molecular Characterization of Geranyl Diphosphate Synthase (GPPS) Genes in Wintersweet Flower

**DOI:** 10.3390/plants9050666

**Published:** 2020-05-24

**Authors:** Hafiz Muhammad Kamran, Syed Bilal Hussain, Shang Junzhong, Lin Xiang, Long-Qing Chen

**Affiliations:** 1 Key Laboratory of Horticultural Plant Biology (Ministry of Education), College of Horticulture & Forestry Sciences, Huazhong Agricultural University, Wuhan 430070, China; silversand15@gmail.com (H.M.K.); bilal.hussain124@yahoo.com (S.B.H.); shangjz@webmail.hzau.edu.cn (S.J.); 2 Southwest Engineering Research Center for Landscape Architecture (State Forestry and Grassland Administration), Southwest Forestry University, Kunming 650224, China

**Keywords:** wintersweet, floral fragrance, GPPS, GGPPS, yeast two-hybrid assay

## Abstract

Geranyl diphosphate synthase (GPPS) is a plastid localized enzyme that catalyzes the biosynthesis of Geranyl diphosphate (GPP), which is a universal precursor of monoterpenes. Wintersweet (*Chimonanthus praecox* L.), a famous deciduous flowering shrub with a strong floral scent character, could have GPPS-like homologs that are involved in monoterpenes biosynthesis, but it remains unclear. In the present study, five full-length GPPS and geranylgeranyl diphosphate synthases (GGPPS) genes were identified in the wintersweet transcriptome database. The isolated cDNAs showed high protein sequence similarity with the other plants GPPS and GGPPS. The phylogenetic analysis further classified these cDNAs into four distinct clades, representing heterodimeric GPPS small subunits (SSU1 and SSU2), homodimeric GPPS, and GGPPS. Analysis of temporal expression revealed that all genes have the highest transcript level at the full-open flower stage. From tissue-specific expression analysis, *CpGPPS.SSU1* and *CpGGPPS1* were predominantly expressed in petal and flower, whereas *CpGPPS.SSU2*, *GPPS*, and *GGPPS2* showed a constitutive expression. Additionally, the subcellular localization assay identified the chloroplast localization of SSUs and GGPPSs proteins, and the yeast two-hybrid assay showed that both *Cp*GPPS.SSU1 and *Cp*GPPS.SSU2 can interact with the GGPPS proteins. Taken together, these preliminary results suggest that the heterodimeric GPPS can regulate floral scent biosynthesis in wintersweet flower.

## 1. Introduction

The floral scent is one of the main features that define the aesthetic value of cut flowers and ornamental plants [1]. It is made up of the specialized metabolites of plants, which enable them to interact with their environment by attracting pollinators and repelling pests, such as herbivores, pathogens, and parasites [2]. The floral scent is the composite of different volatile organic compounds (VOCs), which mainly include terpenoids, phenylpropanoids, benzenoid compounds, and fatty acid derivatives [3]. Moreover, the content and composition of VOCs vary among species and contribute to their distinct fragrance [4]. Overall, terpenoids account for a range of secondary metabolites with numerous volatile constituents, which are derived from two interconvertible five-carbon (C5) precursors (isopentenyl diphosphate (IPP) and its allylic isomer, dimethylallyl diphosphate (DMAPP)) [5]. These C5-isoprene building units are produced from two independent pathways: (1) the mevalonic acid pathway, which leads to the formation of sesquiterpenes; and (2) the methylerythritol phosphate (MEP) pathway, which contributes to the biosynthesis of monoterpenes and diterpenes [6].

In the MEP pathway, DMAPP and IPP molecules are condensed (head to tail) by the geranyl diphosphate synthase (GPPS), resulting in the formation of geranyl diphosphate (GPP) which is a universal precursor of monoterpenes. In several plant species, GPPSs have been characterized as heterodimeric and homodimeric forms [7,8]. Structurally, the heterodimeric GPPS is composed of one large subunit (LSU) and a small subunit (SSU) [9,10,11]. The LSU of heterodimeric GPPS shares high sequence similarity (50–75%) with the geranylgeranyl diphosphate synthases (GGPPS) and possess prenyltransferase activity, producing GGPP and often lower levels of GPP and FPP, while the SSU shares only 22–38% sequence similarity with the GGPPS and have no prenyltransferase activity [9]. The interaction of LSU with SSU causes a shift in the composition and content of the product, which normally favors the formation of GPP as compared to the activity displayed by the LSU alone. The homodimeric GPPS is catalytically active and produces GPP as a product [9,12,13]. Recent studies have shown the involvement of both homodimeric and heterodimeric GPPSs in monoterpenes formation in different plant species, such as *Antirrhinum majus, Humulus lupulus, Clarkia breweri, Phlaenopsis bellina, Arabidopsis thaliana,* and *Mangifera indica* [9,13,14,15,16,17]. Except for monoterpenes, there are also few reports which support the participation of homodimeric GPPS and heterodimeric GPPS in gibberellin and carotenoid biosynthesis in tomato and red pepper, respectively [7,18].

Wintersweet (*Chimonanthus praecox* L.) is a diploid (2n = 22) deciduous flowering shrub and ornamental plant, which belongs to the family Calycanthaceae. It is native to China and has been cultivated as a garden and potted plant for the past 1000 years [19]. Moreover, its unique time to flower (November to March), distinct soothing scent, and prominent yellow color of flower enhance its ornamental values and make it a popular landscape, as well as a cut flower plant [19]. The scent of wintersweet flower is mainly dominated by volatile monoterpenoid, sesquiterpenoid, and benzenoid compounds [20]. However, less is known about their biosynthesis-related molecular mechanism(s). To date, only *CpFPPS* (farnesyl diphosphate synthase) and *CpSAMT* (salicylic acid carboxyl methyltransferase) have been investigated [21,22]. In addition, Tian et al. [23] used transcriptome and proteome approaches to identify potential genes responsible for differences in terpenoids and benzenoid compounds between wintersweet cultivars and suggested that GPPS might play an important role in regulating floral fragrance. 

The present study aimed to screen all the potential unigenes as candidate *Cp*GPPS/GGPPS genes from the transcriptome database of wintersweet plants [23] and further characterized these genes through expression analysis in wintersweet, followed by subcellular localization and protein interaction assays.

## 2. Results

### 2.1. Isolation of cDNAs Encoding CpGPPS/GGPPS

From the floral cDNA library of the *C. praecox* plant, eight cDNAs were found to be annotated as GPPS/GGPPS, of which five cDNAs had full-length open reading frames (ORF). Blast (blastp) results of these ORFs against the NCBI (https://www.ncbi.nlm.nih.gov/), TAIR (https://www.arabidopsis.org/index.jsp), and Sol genomics (https://solgenomics.net/) databases showed that three cDNAs (c81471, c64849, and c90672) have high sequence similarity with the known GPPS.SSU1, GPPS.SSU2, and homodimeric GPPS proteins from other plants, respectively (Table S1). Moreover, the remaining two cDNA (c80517 and c51170) are similar to the GGPPS protein. 

### 2.2. Sequence Comparison, Gene Structure, and Phylogenetic Analysis

Multiple protein sequence alignment of the GPPS/GGPPS-like genes was performed with the MEGA 7 software. The results showed that the amino acid sequence of c90672, c80517, and c51170 contain the two highly conserved aspartate-rich regions [(FARM (DDX_2–4_D) and SARM (DDxxD)] (Figure S1C,D; Table 1), while c64849 has only a FARM motif (Figure S1B; Table 1). Moreover, the amino acid sequence of c81471 has none of these substrate binding motifs (Figure S1A; Table 1). Further comparison identified the two conserved CxxxC motifs in both c81471 and c64849 and one such motif in c80517 and c51170 similar to other plants (Figure S1A,B,D; Table 1). However, c90672 is free from the CxxxC motif, similar to other characterized homomeric GPPSs (Figure S1C; Table 1).

**Figure 1:**
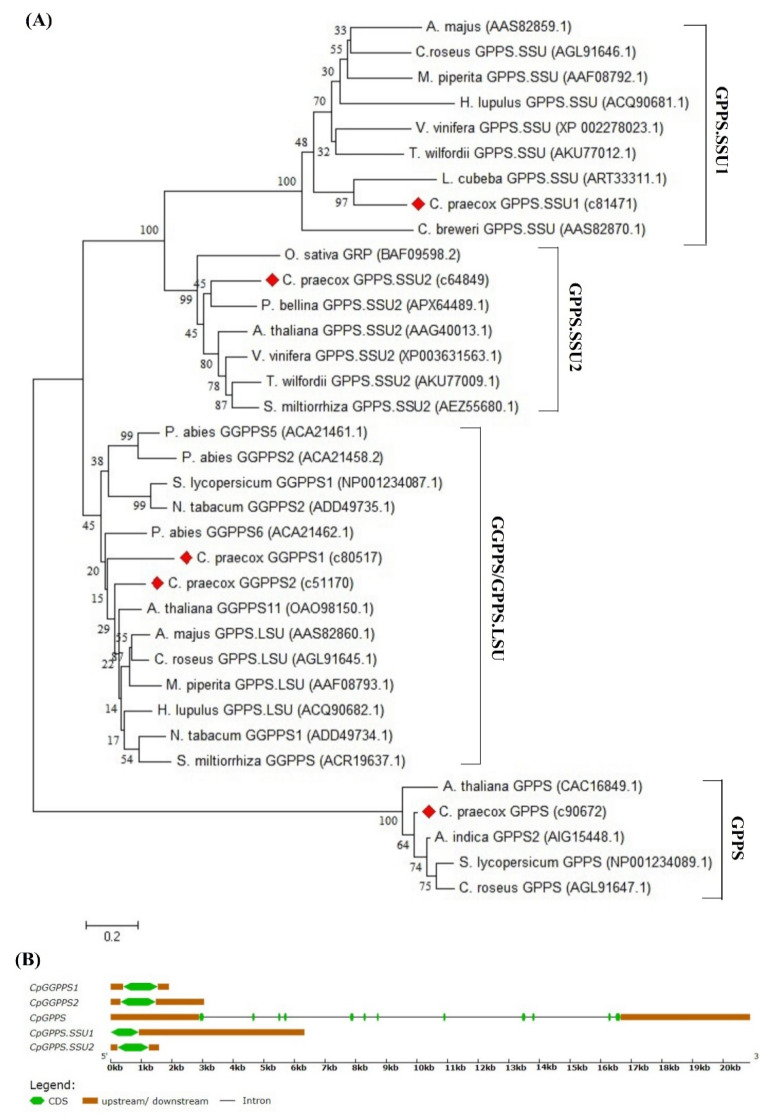
Phylogenetic and gene structure analysis of the wintersweet geranyl diphosphate synthase (GPPS)/geranylgeranyl diphosphate synthase (GGPPS). (**A**) The phylogenetic tree was generated by the neighbor-joining method using the MEGA 7.0, after alignment of deduced amino acid sequences by ClustalW. Numbers in the nodes are the bootstrap support values from 1000 replicates. The accession numbers are given in parenthesis. (**B**) Schematic genomic structure of *Cp*GPPS/GGPPS genes. The green box represents the exon and the black lines between them denote intron. The dark orange box represents the untranslated region.

For evolutionary relationship analysis, GPPS/GGPPS sequences from various plant species were retrieved and used to build the phylogenetic tree. *Cp*GPPS/GGPPS genes were classified into the four distinct clades, representing GPPS.SSU1, GPPS.SSU2, homodimeric GPPS, and GGPPS (Figure 1A). Moreover, GPPS.SSU1, GPPS.SSU2 and homodimeric GPPS contained one gene in each clade, while the GGPPS clade contained two genes (c80517 and c51170). Gene structure analysis displayed that c81471, c64849, c80517, and c51170 are intron-free genes, while c90672 contains 12 exons and 11 introns (Figure 1B). Based on their molecular characteristics these sequences were named *CpGPPS.SSU1*, *CpGPPS.SSU2*, *CpGPPS*, *CpGGPPS1*, and *CpGGPPS2* (Table 1).

The ORFs of *CpGPPS.SSU1*, *CpGPPS.SSU2*, *CpGPPS*, *CpGGPPS1*, and *CpGGPPS2* encode predicted proteins of 306, 341, 428, 377, and 384 amino acids with a molecular weight of 33.00 kDa, 37.60 kDa, 46.84 kDa, 40.85 kDa, and 41.39 kDa and theoretical isoelectric point (pI) of 7.07, 5.75, 5.85, 6.47, and 5.97, respectively (Table 1). 

### 2.3. Spatiotemporal Expression Pattern of CpGPPS/GGPPS Genes

**Figure 2:**
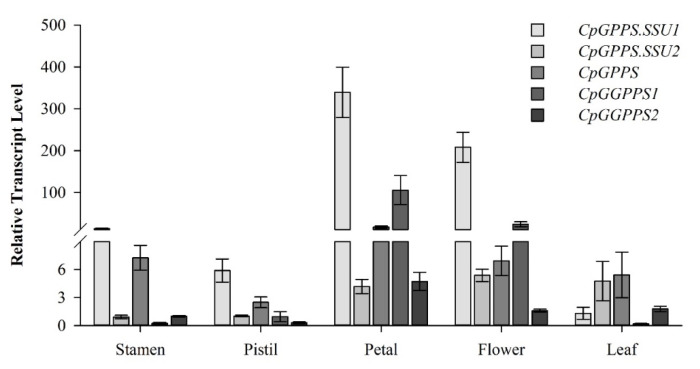
Spatial expression profiles of *CpGPPS.SSU1*, *CpGPPS.SSU2*, *CpGPPS*, *CpGGPPS1*, and *CpGGPPS2* in the wintersweet plant. *β*-Actin was used as a housekeeping gene to normalize gene expression.

In plants, the formation of VOCs is a spatially and developmentally regulated phenomenon [4,24,25]. The transcript level of *Cp*GPPS/GGPPS genes was examined using real-time quantitative polymerase chain reaction (qRT-PCR) in various plant tissues, including the stamen, pistil, petal, flower, and leaf. Relative expression levels of all the *Cp*GPPS/GGPPS genes were compared with reference to the relative expression of *CpGGPPS2* in the stamen. All the *Cp*GPPS/GGPPS genes showed variation in the spatial expression intensities. Among the five genes, *CpGPPS.SSU1* and *CpGGPPS1* showed the highest transcript, followed by *CpGPPS*, *CpGPPS.SSU2*, and *CpGGPPS2*. In detail, *CpGPPS.SSU1* and *CpGGPPS1* genes were highly expressed in the petal and flower but had low expression levels in the leaf, pistil, and stamen. Moreover, *CpGPPS.SSU2*, *CpGPPS*, and *CpGGPPS2* were expressed in all the examined tissues, with high transcript levels in the petal (Figure 2).

**Figure 3:**
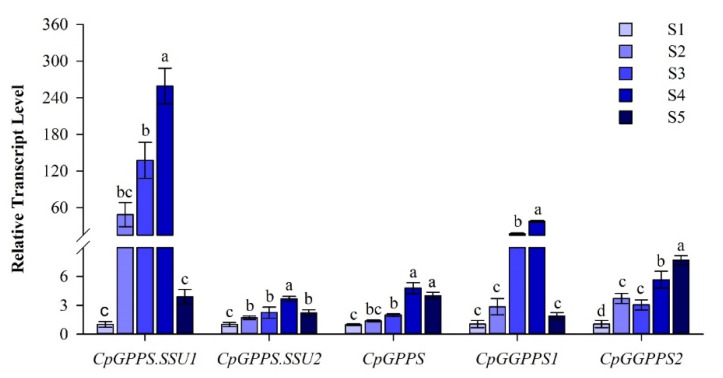
Temporal expression profile of *CpGPPS.SSU1*, *CpGPPS.SSU2*, *CpGPPS*, *CpGGPPS1*, and *CpGGPPS2* in different developmental stages of the wintersweet flower. Different lowercase letters on bars indicate significant differences among tissues at *p* < 0.05 based on HSD post-hoc test. S1: Closed bud with green petals, S2: Closed bud with yellow petals, S3: Partial open flower, S4: Open flower but not pollinated, S5: Senescent flower.

The temporal expression analysis of *Cp*GPPS/GGPPS genes in flowers revealed that all genes had the lowest expression levels in stage 1 (Figure 3). Moreover, the expression levels of all *Cp*GPPS/GGPPS genes, except *CpGGPPS2* genes, increased gradually and reached a peak in stage 4, whereas their expression levels were significantly decreased in stage 5 (Figure 3). Interestingly, a prominent change in the temporal expression profile was observed for *CpGPPS.SSU1* and *CpGGPPS1* genes. Their level of expression was significantly increased in stage 4, which was approximately 245- and 35-time higher than that of stage 1, respectively (Figure 3). 

### 2.4. Subcellular Localization of CpGPPS/GGPPS Genes

**Figure 4:**
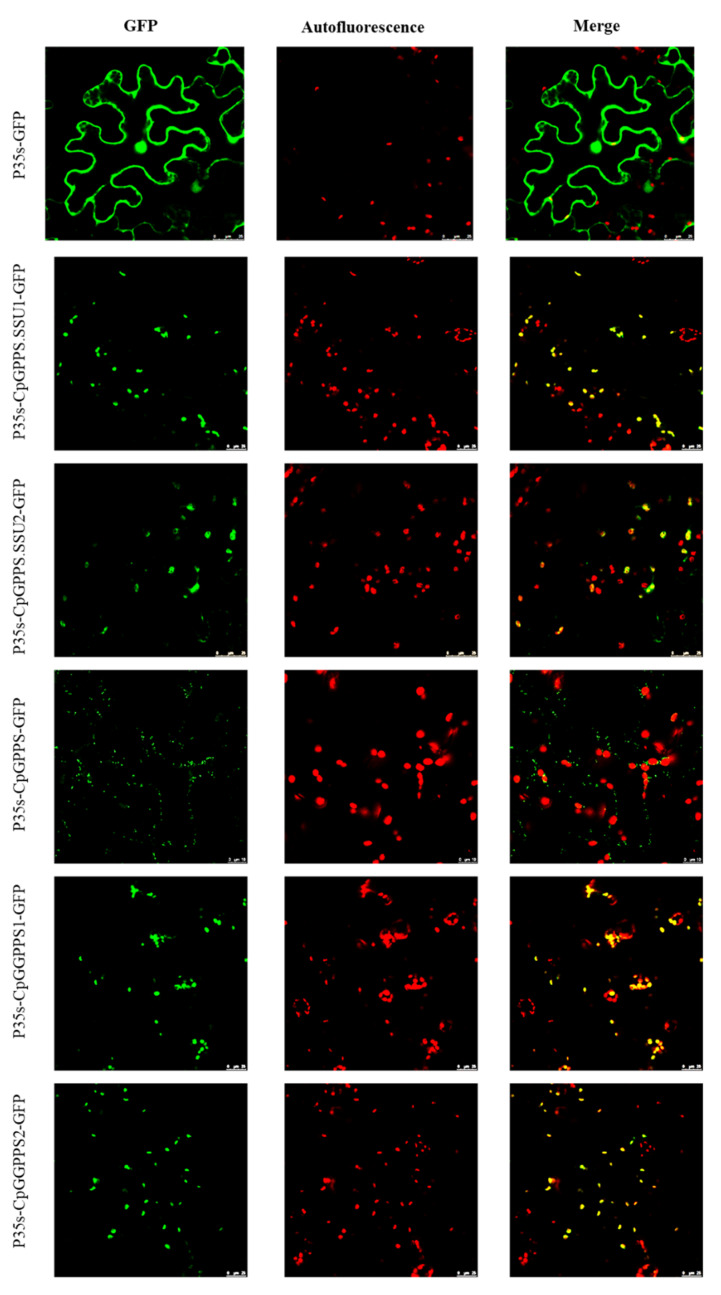
Subcellular localization of *Cp*GPPS.SSU1, *Cp*GPPS.SSU2, *Cp*GPPS, *Cp*GGPPS1, and *Cp*GGPPS2 in the *N. benthamiana* leaves. GFP signals from the adaxial leaf surface were observed by using a confocal laser scanning microscope. GFP, green fluorescence image; Autofluorescence, chlorophyll autofluorescence image; Merge, overlay of GFP and autofluorescence.

In silico study indicated the presence of putative transit peptide in *Cp*GPPS.SSU1, *Cp*GPPS.SSU2, *Cp*GGPPS1, and *Cp*GGPPS2 proteins with the chloroplast localization, while the *Cp*GPPS protein was found to be localized in the mitochondria (Table 1). To validate, protein sequences of *Cp*GPPS.SSU1, *Cp*GPPS.SSU2, *Cp*GGPPS1, *Cp*GGPPS2, and *Cp*GPPS were fused to the N-terminus of the PRI101-EGFP vector, and these recombinant vectors were transiently expressed into the *Nicotiana benthamiana* leaves. Through microscopy, the green fluorescent protein (GFP) fluorescence signals of *Cp*GPPS.SSU1, *Cp*GPPS.SSU2, *Cp*GGPPS1, and *Cp*GGPPS2 were detected in the chloroplasts, whereas *Cp*GPPS-GFP fusion protein fluorescence signals were detected in the mitochondria (Figure 4).

### 2.5. CpGPPS.SSU1 and CpGPPS.SSU2 Interaction with GGPPS

**Figure 5:**
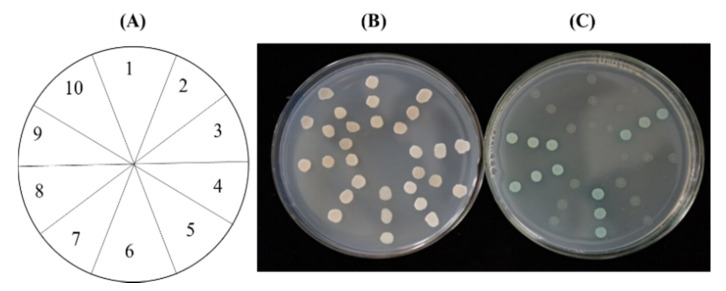
Interaction of *Cp*GPPS.SSUs with *Cp*GGPPSs and *At*GGPPS11. (**A**) Key: 1, BD-*Cp*SSU1 + AD-empty; 2, BD-*Cp*SSU1 + AD-*Cp*GGPPS1; 3, BD-*Cp*SSU1 + AD- *Cp*GGPPS2; 4, BD-*Cp*SSU1 + AD-*At*GGPPS11; 5, BD-Lam + AD-Rec-T (negative control); 6, BD-53 + AD-Rec-T (positive control); 7, BD-*Cp*SSU2 + AD-*Cp*GGPPS1; 8, BD-*Cp*SSU2 + AD-*Cp*GGPPS2; 9, BD-*Cp*SSU2 + AD- *At*GGPPS11; 10, BD-*Cp*SSU2 + AD-empty. (**B**) The *Cp*GPPS.SSUs were fused with the DNA binding domain (BD) of PGBKT7 and GGPPSs were fused with the activation domain (AD) of the PGADT7 vector. Yeast strain AH109 co-transformed pairwise with both binding and activation vectors and spotted on the minimal synthetic dropout (SD) medium without leucine and tryptophan. (**C**) Yeast strains relative to those in the left panel were spotted on the SD medium lacking adenine, histidine, leucine, and tryptophan supplied with X-α-Gal.

To validate if the *Cp*GPPS.SSU1 and *Cp*GPPS.SSU2 can interact with the GGPPS/GPPS.LSU to form a heterodimer, we performed the yeast 2 hybrid (Y2H) assay. The result revealed that yeast cells carrying the plasmid pairs BD-*Cp*GPPS.SSU1 + AD-*Cp*GGPPS2, BD-*Cp*GPPS.SSU2 + AD-*Cp*GGPPS2, BD-*Cp*GPPS.SSU2 + AD-*At*GGPPS11 and the positive control plasmid pair grew and turned blue on the QDO/X medium, indicating that the testing plasmid pair could interact and activate the reporter genes in the GAL4 system. On the other hand, yeast cells harboring the plasmid pairs, namely BD-*Cp*GPPS.SSU1 + AD-*Cp*GGPPS1, BD-*Cp*GPPS.SSU2 + AD-*Cp*GGPPS1, BD-*Cp*GPPS.SSU1 + AD-*At*GGPPS11, BD-*Cp*GPPS.SSU1 + AD-empty, and BD-*Cp*GPPS.SSU2 + AD-empty plasmid pairs and negative control could not grow and did not show any interaction on the QDO/X medium (Figure 5).

## 3. Discussion

Short-chain prenyl diphosphate synthases are enzymes of the isoprenoid pathway that use IPP and DMAPP to produce central intermediates in the isoprenoid metabolism. Short-chain prenyl diphosphate synthases are represented by three enzymes: GPPS, FPPS, and GGPPS [26]. Isolation and functional characterization of GPPSs from different plant species have confirmed the existence of homodimeric and heterodimeric forms, which are comprised of two similar subunits and one GPPS.SSU with one GPPS.LSU, respectively [15]. From the wintersweet transcriptome database [23], we isolated five transcripts that were annotated as GPPS/GGPPS-like genes: one homodimeric GPPS (*CpGPPS*), two GPPS small subunits (*CpGPPS.SSU1*, *CpGPPS.SSU2*), and two GGPPS (*CpGGPPS1*, and *CpGGPPS2*) sequences (Table 1). Subsequent sequence analysis indicated that homodimeric *Cp*GPPS and both *Cp*GGPPSs have two conserved aspartate-rich motifs, containing FARM (DD(X)_2–4_D) and SARM (DDXXD) (Figure S1C,D; Table 1), which are essential for catalysis and substrate binding [27]. However, *Cp*GPPS.SSUs lack one or both of these motifs (Figure S1A,B; Table 1). Besides, the CxxxC motif is needed for physical interaction between both subunits of heterodimeric GPPS [13,14,15]. In the present study, two CxxxC motifs were found in *Cp*GPPS.SSUs and one motif in *Cp*GGPPSs (Figure S1A,B,D; Table 1), which is consistent with the previous findings from other plant species.

The scent of the wintersweet flower is composed of different volatile compounds, of which α-linalool is the most abundant monoterpene volatile compound, accounting for about 36% of the wintersweet floral scent [28]. Moreover, the formation of monoterpenes possesses tissue specificity and is often related to the maturity of a given tissue [29,30]. The wintersweet flower develops a strong and specific fragrance during flower opening [31]. Further, wintersweet flowers contain the maximum number of scent-emitting nectaries at the fully opened flower stage. Dominantly, the maximum number of scent-emitting nectaries resides on petals during flower development [32]. In the present study, *Cp*GPPS/GGPPS genes were expressed in all tested tissues, with varying levels (Figure 2). Namely, the *CpGPPS.SSU1* and *CpGGPPS1* were highly expressed in the petal and flower, suggesting that these genes could have an important role in scent-producing tissues. Temporal expression analysis revealed that all *Cp*GPPS/GGPPS genes, except *CpGGPPS2*, showed the maximum expression at fully opened flower stage, followed by reduction at the senescence stage (Figure 3). These expression profiles are consistent with the finding of Xiang et al. [21], who reported that the *C. praecox* L. ‘H29’ flowers had the maximum monoterpene contents at the partially opened (58.06%) and fully opened stage flowers (57.89%), followed by a sudden decline at senescence (9.44%). Overall, these results suggest that the spatiotemporal expression of *CpGPPS.SSU1* and *CpGGPPS1* is strictly confined to the scent-emitting tissues with a good association to monoterpene contents. Moreover, Chen et al. (2015) reported the constitutive expression of *AtGPPS.SSU2* and found its involvement in monoterpene biosynthesis [16]. This finding indicates that we cannot neglect the role of *CpGPPS.SSU2* in scent biosynthesis in the wintersweet flower and suggest further investigations. 

All genes/enzymes involved in the MEP pathway are known to have a transit peptide for their plastid targeting. The subcellular localization experiments in *Catharanthus roseus* and *Litsea cubeba* displayed the plastidial localization of GPPS/GGPPS [12,18,33]. On the other hand, there are few reports which support the mitochondrial, cytosolic, and endoplasmic reticulum localization of this pathway’s genes [26]. In the present study, the subcellular localization assay revealed that *Cp*GPPS.SSU1, *Cp*GPPS.SSU2, *Cp*GGPPS1, and *Cp*GGPPS2 proteins have strong GFP signals in the chloroplast region similar to the previous findings of GPPS.SSU1 from *A. majus,* GPPS.SSU2 from *C. annuum* and GGPPSs from *A. thaliana* [14,26]. However, the GFP fluorescence of *Cp*GPPS protein was not observed in the chloroplast, likely because it is localized in the mitochondria (Figure 4), consistent with the previous result of homodimeric GPPS in *C. roseus* [12]. These results support the chloroplast localization of *CpGPPS.SSU1* and *CpGPPS.SSU2* genes, further suggesting the functional involvement of these genes in monoterpene biosynthesis and metabolism. 

In the heterodimeric GPPS gene, LSU is active and functional itself, while SSU is inactive and needs to interact with the LSU for functioning [9,12]. Through Y2H assay, both the *Cp*GPPS.SSU1 and *Cp*GPPS.SSU2 showed interaction with *Cp*GGPPS2. Moreover, *Cp*GPPS.SSU2 also interacted with the *At*GGPPS11 (Figure 5). These results support the possibility of heterodimeric GPPS existence in the wintersweet plant and warrant speculation that the SSU of heterodimeric GPPS could have a role in modifying or accelerating the product specificity of LSU or GGPPS. These assertions are consistent with previous suggestions. For instance, following the bacterial genetic complementation assay, when *Li*GPPS.SSU1 and *Li*GPPS.SSU2 were individually co-transformed with catalytically active *LiGGPPS*, both SSU interacted and reduced the carotenoid contents [34]. Moreover, when *AmGPPS.SSU1* was transformed in tobacco, it act as a modifier and increases the GPP, as well as monoterpene biosynthesis, at the expense of GGPP-derived compounds, i.e., gibberellins, chlorophyll, and carotenoids [14]. On the other hand, *MpGPPS.SSU1* act like an accelerator when overexpressed in tobacco, as it promotes shoot branching and early flowering by an elevated content of cytokinin and gibberellin. Wang et al. [18] reported that the virus-induced gene silencing of *CaSSU2* led to a reduction in carotenoid content in red pepper fruit. However, the knowledge about this phenomenon remains unknown in wintersweet. In the future, all *Cp*GPPS/GGPPS candidate genes need to be further investigated by exploiting techniques such as *in vitro* enzymatic assays and genetic transformation to obtain deeper insights.

## 4. Materials and Methods

### 4.1. Plant Material and Sampling

**Figure 6:**
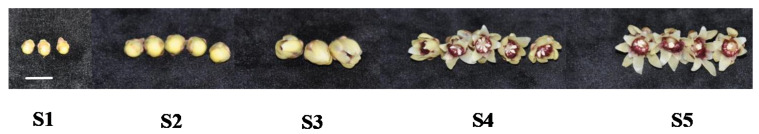
Flower development stages in wintersweet. **S1**: Closed bud with green petals, **S2**: Closed bud with yellow petals, **S3**: Partial open flower, **S4**: Open flower but not pollinated, **S5**: Senescent flower. Scale bar = 1 cm.

A healthy and vigorous wintersweet plant (*C. praecox* L. ‘H29’) was selected as the study material. The experimental plant was grown under natural field conditions in the campus of Huazhong Agricultural University (Wuhan, China). Flower samples were collected at five developmental stages (stage 1 to stage 5), from bud development to senescence (Figure 6). In detail, flower developmental stages were characterized with the following distinct morphological characters: Closed bud with green petals (stage 1), closed bud with yellow petals (stage 2), partial open flower (stage 3), open flower but not pollinated (stage 4), and senescent flower (stage 5). For spatial expression analysis, the leaf, open flower, stamen, pistil, and petal samples were collected. All samples were immediately frozen in liquid nitrogen and stored at −80 °C for further use.

### 4.2. Database Mining and Sequencing of CpGPPS cDNAs

The GPPS/GGPPS sequences of *A. thaliana* and *L. cubeba* were used as a query to blast against the *C. praecox* genome database (unpublished), and the floral transcriptome database [23] to retrieve all the putative *Cp*GPPS/GGPPS protein sequences from wintersweet. A blastp analysis of the retrieved *Cp*GPPS/GGPPS protein sequences was carried out by searching against the NCBI (https://www.ncbi.nlm.nih.gov/), Sol genomics (https://solgenomics.net/), and TAIR (https://www.arabidopsis.org/index.jsp) databases to find their homologs in other plant species. The largest ORF of *Cp*GPPS/GGPPSs were amplified using the high-fidelity DNA polymerases (Vazyme, China) with gene-specific primers (Table S2). PCR was performed in a 50-µL reaction mixture comprising phanta max superfidelity DNA polymerases (11.5 μL, 1 unit/µL), forward and reverse primer (each 2.5 μL, 10 µM/µL), cDNA template (2 μL, 1 µg/µL) and ddH_2_O (31.5 μL) was subjected to the following PCR instructions: 95 °C for 5 min, and 35 cycles of 95 °C for 30 s, 60 °C for 30 s, 72 °C for 45 s, and a final extension of 72 °C for 7 min. The amplicons were purified using the Tiangel Midi Purification Kit (Tiangen, China) and cloned into a pEASY-T1 cloning vector (Transgen, China) for sequencing. The consistent sequencing results of each *Cp*GPPS/GGPPSs were used for subsequent analysis.

### 4.3. Bioinformatics Analysis

The ORF of *Cp*GPPS/GGPPSs were identified using the online tool ‘ORF finder’ (http://www.ncbi.nlm.nih.gov/gorf/gorf.html). Molecular weight and pI values of the putative proteins were predicted with the ExPASy tool (http://web.expasy.org/compute_pi/). The NCBI conserved domain tool (https://www.ncbi.nlm.nih.gov/Structure/cdd/wrpsb.cgi) was used to identify the functional domains. TargetP 1.1 server (http://www.cbs.dtu.dk/services/TargetP/), ChloroP (http://www.cbs.dtu.dk/services/ChloroP/) and PSORT (http://psort.hgc.jp/) systems were used to predict the signal peptides and subcellular localization. Multiple sequence alignment of *Cp*GPPS/GGPPS sequences, as well as the phylogenetic tree, was constructed using the MEGA 7 software, following the neighbor-joining method [35]. Gene structure was predicted using an online GSDS tool (http://gsds.cbi.pku.edu.cn/; Hu, et al. [36]).

### 4.4. RNA Extraction and qRT-PCR

Total RNA was extracted from different samples using the Hipure plant RNA purification kit (Magen Biotech, China), following the manufacturer’s instructions. One microgram of high-quality RNA was used to synthesize the first strand of cDNA using TransScript One-Step gDNA Removal and cDNA Synthesis SuperMix (TransGen Biotech, China). qRT-PCR was performed in the 10-µL reaction mixture comprising 5 µL of SYBR^®^ Premix Ex Taq™ II mix (Takara, Dalian, China), 0.3 µL of primer (forward and reverse each; the final concentration of 10 µM), 3.9 µL of ddH_2_O, and 0.5 µL of cDNA template with a final concentration of 300 ng/µL. The reactions were carried out using a Quant studio 7 flex real-time system (Applied Biosystems Life Technologies, New York), following the manufacturer’s protocol. The reaction was started with an initial incubation at 50 °C for 2 min and 95 °C for 5 min, then subjected to 40 cycles of 95 °C for 10 s, 60 °C for 20 s, and 72 °C for 20 s. qRT-PCR was performed in three biological replicates, and each biological replicate consisted of three technical replicates. Moreover, β-actin was used as a housekeeping gene to normalize the relative expression of target genes. Relative gene expression was analyzed following the 2^–ΔΔCT^ method [37].

### 4.5. Subcellular Localization 

For protein localization, we generated the green fluorescent fusion protein in frame with *Sma1*/*EcoR1* cloning sites of the PRI101-EGFP vector driven by CaMV 35S promoter. The coding sequence of *Cp*GPPS/GGPPSs were amplified (without stop codon) from *C. praecox* cDNA using gene-specific primers (Table S2). Amplified fragments were fused with the 5′ end of the GFP sequence to generate a cassette comprising of 35S-*Cp*GPPSs-EGFP. The resulting constructs of *Cp*GPPSs-EGFP fusion were sequenced to confirm the protein fusions. All the constructs were then transferred into *A. tumefaciens* strain (GV3101+p19) by a freeze-thaw method. Transformed Agrobacterium clone was grown in LB medium until the OD_600_ value reached 0.4–0.6 and was then used to infiltrate the leaves of 3–5-week-old *N. benthamiana* plants. The suspension solution was infiltrated on the lower surface of leaves using a 1-mL sterile needleless syringe, followed by a dark period for two days [38]. After 2–3 days of infiltration, the fluorescence signal of the GFP was examined at 488 nm under a confocal microscope (TCS-SP8, Leica, Germany).

### 4.6. Yeast 2-Hybrid Assay (Y2H)

The truncated versions of *Cp*GPPS.SSU1, *Cp*GPPS.SSU2, *Cp*GGPPS1, *Cp*GGPPS2, and *At*GGPPS11 without the predicted plastid target peptide were amplified and sequenced using the gene-specific primers (Table S2). The amplified fragments of *Cp*GPPS.SSU1 and *Cp*GPPS.SSU2 were cloned in frame with the binding domain (GAL4) of the pGBKT7 (bait) vector, while *Cp*GGPPS1, *Cp*GGPPS2, and *At*GGPPS11 were introduced in frame with the activation domain (GAL4) of the PGADT7 (prey) vector to use in protein interaction evaluation. The respective pairs of bait and prey vectors were co-transformed into the yeast strain (AH109) by the LiAc/DNA/PEG method, following the Yeast Protocols Handbook from Clontech (http://www.clontech.com). Co-transformed AH109 cells were cultured on the synthetic dropout (SD) media without Leu and Trp, and the interaction/activation of two proteins was studied on the SD media lacking Leu, Trp, His, and Ade.

### 4.7. Statistical Analysis

The data were evaluated by the HSD post-hoc test in the ANOVA program of Statistix 8.1 (Florida, USA). Different lowercase letters on bars indicate significant differences at *p* < 0.05.

## 5. Conclusions

Five GPPS/GGPPS genes (*Cp*GPPS.SSU1, *Cp*GPPS.SSU2, *Cp*GPPS, *Cp*GGPPS1, and *Cp*GGPPS2) were isolated in wintersweet flower. qRT-PCR results showed that the expression of *CpGPPS.SSU1* was predominantly confined to the fragrance-producing tissues and was upregulated during flower development, indicating important roles in floral volatile emission. In addition, the present data confirm that both *Cp*GPPS.SSUs can interact with GGPPS-like proteins, and localization experiments further support the presence of chloroplast localized heterodimeric GPPS in the wintersweet flower. Collectively, the present data warrant consideration of heterodimeric GPPS.SSU as a target for the manipulation of floral scent production. Further studies will better define the modes of regulation and confirm the roles of heterodimeric GPPS in wintersweet flower scent production.

## Figures and Tables

**Table 1:** Characteristics of *Cp*GPPS/GGPPS genes.

Gene/cDNA Name	ORF Length (aa)	Theoretical pI Value	Molecular Weight (kDa)	In Silico Subcellular Localization Prediction	Conserved Motif
*CpGPPS.SSU1*/c81471	306	7.07	33.00	Chloroplast	CxxxC, CxxxC
*CpGPPS.SSU2*/c64849	341	5.75	37.60	Chloroplast	CxxC, DDX(_2–4_)D, CxxxC
*CpGPPS*/c90672	428	5.85	46.84	Mitochondria	DDX(_2–4_)D, DDxxD
*CpGGPPS1*/c80517	377	6.47	40.85	Chloroplast	CxxxC, DDX(_2–4_)D, DDxxD
*CpGGPPS2*/c51170	384	5.97	41.39	Chloroplast	CxxxC, DDX(_2–4_)D, DDxxD

## Supplementary Materials

The following are available online at https://www.mdpi.com/2223-7747/9/5/666/s1, Table S1: Protein sequence homology of *Cp*GPPS/GGPPSs. Table S2: List of primers used in this study. Figure S1: Multiple sequence alignment of deduced amino acid of GPPS.SSU1 (A), GPPS.SSU2 (B), homodimeric GPPS (C) and GGPPS (D) like proteins from wintersweet.
