# Supplementary material for: Identification and Molecular Characterization of Geranyl Diphosphate Synthase (GPPS) Genes in Wintersweet Flower
Source: Plants (Basel). 2020 May 24;9(5):666. doi: 10.3390/plants9050666 (PMC7284688; doi:10.3390/plants9050666)
Supplement: Supplementary file 1 [file plants-09-00666-s001.pdf]

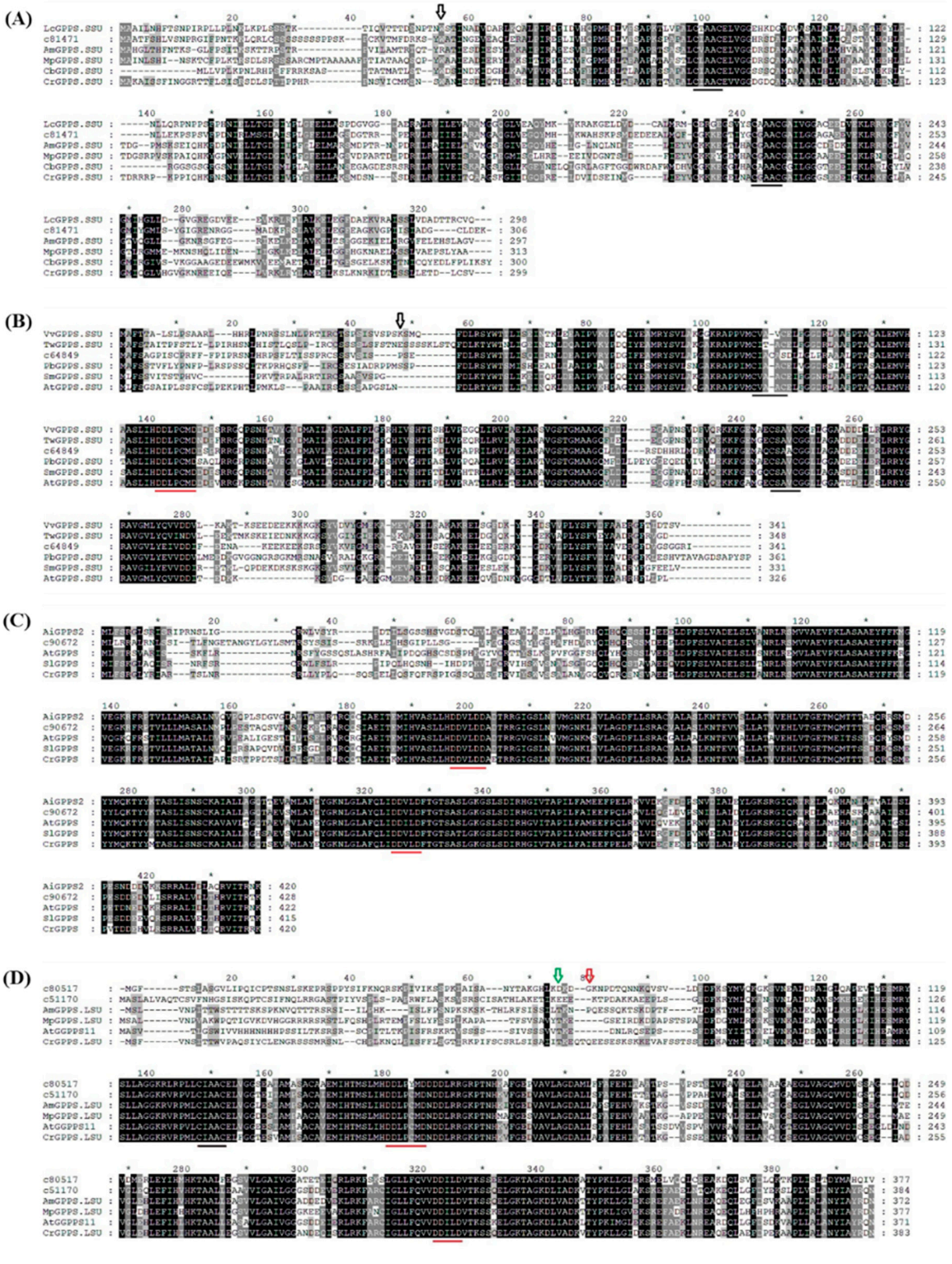

Figure S1. Multiple sequence alignment of deduced amino acid of GPPS.SSU1 (**A**), GPPS.SSU2 (**B**), homodimeric GPPS (**C**) and GGPPS (**D**) like proteins from wintersweet and other plants. Identical amino acids are shaded, and gaps are indicated by dots. The conserved aspartate-rich region motif $DDX_{2-4}D$ (Red solid line) and CxxxC motif (black solid line) are underlined. The truncation of *Cp*GPPS.SSU1 and *Cp*GPPS.SSU2 are marked with black arrowhead, while *Cp*GGPPS1 and *Cp*GGPPS2 were marked with green and red arrowheads, respectively.

Table S1: Protein sequence homology of *Cp*GPPS/GGPPS

Gene	Sequence Similarity
CpGPPS.SSU1	*Litsea cubeba* 54.97%,<br>*Quercus lobate* 48.71%,<br>*Antirrhinum majus* 43.96%
CpGPPS.SSU2	*Populus trichocarpa* 62.61%,<br>*Salvia miltiorrhiza* 64.09%
CpGGPPS2	*Litsea cubeba* GGPPS 79.17%,<br>*Humulus lupulus* GPPS.LSU 69.02%
CpGGPPS1	*Litsea cubeba* GGPPS 60.94%,<br>*Humulus lupulus* GPPS.LSU 65.02%
CpGPPS	*Cathaeanthus roseus* GPPS 80.48%,<br>P*hoenix dactylifera* 79.72%

Table S2: List of primers used in this study

Gene	Function	Primer	
		Forward Primer (5' to 3')	Reverse Primer (5' to 3')
CpGPPS.SSU1	Gene Cloning	ATGGCAGCAACTTTCAGTCATTTG	TTATTTCTCATCCAAGCACCCATCT
	Real-Time PCR	GAGGTGGAGAAACTGAGGAGGTA	ATCTGCAATACTGATGATGGGAC
	Localization Study	cccgggATGGCAGCAACTTTCAGTCATTTG	gaattcTTTCTCATCCAAGCACCCATCT
	Y2H Study	catatgTGGGCGACCATAAATGGAG	gaattcTTATTTCTCATCCAAGCACCCATC
CpGPPS.SSU2	Gene Cloning	ATGGCCTTCTCAGCAGGACCT	TCAAATTCTCCCACCACTCCCA
	Real-Time PCR	CGAGAAGGCGAAGAAGGAAT	CTCCCACCACTCCCAACATC
	Localization Study	cccgggATGGCCTTCTCAGCAGGACCT	gaattcAATTCTCCCACCACTCCCA
	Y2H Study	gaattcCCATCTGAGTTCGACCTCCGA	ggatccTCAAATTCTCCCACCACTCCC
CpGGPPS1	Gene Cloning	ATGGGTTTTTCGACGAGTTTGG	TTAAACAATTTGGTGGGCCATG
	Real-Time PCR	TCTTCTCCAAAGATCGCCATTTC	CAGTCCGATTGCCCTGTCCA
	Localization Study	cccgggATGGGTTTTTCGACGAGTTTGG	gaattcAACAATTTGGTGGGCCATG
	Y2H Study	gaattcGGAAAAAACCCAGATACCCAA	ggatccTTCCCTTCTGGACCATGTAGC
CpGGPPS2	Gene Cloning	ATGGCTTCACTTGCTTTGGT	CCATTTGGCTATCTAACAGGAAC
	Real-Time PCR	ACCCATCTATGTTTCCCCCC	TCCAGAAAGGGGTTCTTCCG
	Localization Study	cccgggATGGCTTCACTTGCTTTGGT	gaattcGTTCTGTCTGTAAGCAATATA
	Y2H Study	gaattcGAAGAAGAGAAAACCCCAGATGC	ggatccCCATTTGGCTATCTAACAGGAACA
CpGPPS	Gene Cloning	ATGCTTCTGAGACGGGCC	CTAAGAAATAAAACATGGAATAGAA
	Real-Time PCR	GGTTTTTGGTGGCAGAGGAAG	CAGCAACCACCATTGACCGC
	Localization Study	cccgggATGCTTCTGAGACGGGCC	gaattcCTCTATTGCAAATAATATTGGAGC
